# The Role of the Gut Microbiome in Resisting Norovirus Infection as Revealed by a Human Challenge Study

**DOI:** 10.1128/mBio.02634-20

**Published:** 2020-11-17

**Authors:** N. V. Patin, A. Peña-Gonzalez, J. K. Hatt, C. Moe, A. Kirby, K. T. Konstantinidis

**Affiliations:** a School of Biological Sciences, Georgia Institute of Technology, Atlanta, Georgia, USA; b Max Planck Tandem Group in Computational Biology, Department of Biological Sciences, Universidad de los Andes, Bogotá, Colombia; c School of Civil and Environmental Engineering, Georgia Institute of Technology, Atlanta, Georgia, USA; d Rollins School of Public Health, Emory University, Atlanta, Georgia, USA; e Waterborne Disease Prevention Branch, Centers for Disease Control and Prevention, Atlanta, Georgia, USA; Michigan State University; University of Pittsburgh

**Keywords:** human microbiome, metagenomics, noroviruses

## Abstract

Norovirus infections take a heavy toll on worldwide public health. While progress has been made toward understanding host responses to infection, the role of the gut microbiome in determining infection outcome is unknown. Moreover, data are lacking on the nature and duration of the microbiome response to norovirus infection, which has important implications for diagnostics and host recovery. Here, we characterized the gut microbiomes of subjects enrolled in a norovirus challenge study. We analyzed microbiome features of asymptomatic and symptomatic individuals at the genome (population) and gene levels and assessed their response over time in symptomatic individuals. We show that the preinfection microbiomes of subjects with asymptomatic infections were enriched in *Bacteroidetes* and depleted in *Clostridia* relative to the microbiomes of symptomatic subjects. These compositional differences were accompanied by differences in genes involved in the metabolism of glycans and sphingolipids that may aid in host resilience to infection. We further show that microbiomes shifted in composition following infection and that recovery times were variable among human hosts. In particular, *Firmicutes* increased immediately following the challenge, while *Bacteroidetes* and *Proteobacteria* decreased over the same time. Genes enriched in the microbiomes of symptomatic subjects, including the adenylyltransferase *glgC*, were linked to glycan metabolism and cell-cell signaling, suggesting as-yet unknown roles for these processes in determining infection outcome. These results provide important context for understanding the gut microbiome role in host susceptibility to symptomatic norovirus infection and long-term health outcomes.

## Abstract (precis)

The role of the human gut microbiome in determining whether an individual infected with norovirus will be symptomatic is poorly understood. This study provides important data on microbes that distinguish asymptomatic from symptomatic microbiomes and links these features to infection responses in a human challenge study. The results have implications for understanding resistance to and treatment of norovirus infections.

## INTRODUCTION

Noroviruses are the most common cause of acute gastroenteritis outbreaks in the United States, with approximately 20 million cases reported annually. These RNA viruses of the *Caliciviridae* family are responsible for ∼70,000 hospitalizations and ∼800 deaths in the United States alone and ∼50,000 deaths of children under the age of five worldwide (1). Although the prototype “Norwalk virus” was discovered in 1972 (2), lack of cell culture and animal model systems hampered research into the virus’ mode of infection for several decades. The discovery of the first murine norovirus (3) and, more recently, the development of human cell models (4, 5) have accelerated discoveries of norovirus tropism and pathogenicity. These studies have also implied a significant role of enteric bacteria in host immune response.

Despite norovirus infection prevalence and severity, human challenge studies show certain individuals can resist infection (6, 7), and up to 30% of infected individuals are asymptomatic (8, 9). Susceptibility to infection following norovirus infection is dependent on the histo-blood group antigen (HBGA) profile of the host (7, 10). HBGAs are complex carbohydrates that form the outermost part of glycans or glycolipids found on host cell surfaces, including those of the human small intestinal epithelium where human noroviruses (HuNoVs) initiate infection. The molecular recognition profile is specific to the viral strain, with different strains of prototype HuNoV (Norwalk virus) having different capacities for binding HBGAs (11). Although little is known about HuNoV entry into susceptible target cells, HBGAs act as the attachment receptors to initiate binding of viral particles (10, 12). Individuals expressing HBGAs corresponding to the viral pathotype are susceptible to HuNoV infection (6, 13), likely facilitating viral access to a different range of target intestinal cells, including enterocytes, macrophages, T cells, B cells, and dendritic cells (5, 14–17). However, a proportion of people theoretically susceptible to infection (i.e., those expressing the appropriate oligosaccharides) are resistant (6) and/or show no symptoms (18). This phenomenon suggests multiple factors contribute to the response, including a potential role for bacterial interactions with HuNoV. HBGA-like molecules can also be expressed by some enteric bacteria, mostly Gram-negative *Enterobacteriaceae*; HuNoV binds efficiently to these molecules, possibly facilitating infection by either preventing the removal of the virus through shedding or by absorbing/capturing viral particles, thereby repressing the virus’s ability to establish a lasting infection. The effects of this interaction in the transmission and infection process are yet to be established (14, 19). Microbial compounds localized on or outside the cell surface are collectively referred to as extracellular polymeric substances (EPS) and include glycans, lipids, proteins, and other biomolecules that may interact with viral particles. The full scope of these interactions is unknown, but they are likely a key component of understanding viral-microbiome outcomes. The role of commensal bacteria in facilitating HuNoV infection is further supported by evidence that antibiotic-mediated depletion of mouse intestinal microbiota significantly reduces the level of murine NoV (MuNoV) replication (14, 20). Indeed, a recent report by Madrigal et al. (21) showed that, like HuNoV, murine noroviruses can directly bind to a range of commensal bacteria, including Enterobacter cloacae, Escherichia coli, Pseudomonas aeruginosa, Lactobacillus acidophilus, Lactobacillus gasseri, and Bacteroides dorei, with different affinities with each taxon. Neither bacterial growth phase nor temperature significantly affected the binding capacity. In addition, these authors showed that MuNoV can bind to the human commensal fungus Candida albicans.

Nonetheless, microbe-HuNoV interactions *in situ* and the relationship between microbiome biochemical functions and symptomatic outcomes of HuNoV infections remain poorly understood. Enteric viruses are thought to interact with other molecules commonly expressed on bacterial cell surfaces, particularly glycans such as lipopolysaccharides (22). In mice, vitamin A was found to inhibit MuNoV infection by increasing levels of bacteria in the family *Lactobacillaceae* (23), suggesting a role for those microbes in combating norovirus infections. Antibiotic treatment of mice was found to prevent persistent enteric MuNoV infection, and this effect depended on the presence of an antiviral cytokine, although the exact underlying mechanism is not understood (20). The relevance of these findings for human noroviruses and hosts is also not known.

Viral shedding can occur at high levels regardless of symptomatic status (18, 24, 25), suggesting there are host- and/or microbiome-mediated immune responses that prevent symptoms. Shedding can persist for several weeks even after symptoms subside (8, 24); microbiome changes could therefore also be long lasting. However, human microbiome studies assessing gut microbiome responses over the course of norovirus infection are limited. Even fewer data are available to differentiate the microbiomes of asymptomatic individuals from those of symptomatic individuals. For instance, one 16S rRNA gene amplicon study showed a minority of infected patients featured shifts in their microbiome following infection, but the level of taxonomic resolution provided was poor and changes were inconsistent (26).

In this study, we address these knowledge gaps in norovirus-microbiome interactions by performing a controlled infection study. Subjects ingested HuNoV GI.1 via oysters seeded with viral particles and were placed in either a symptomatic or asymptomatic outcome group depending on the occurrence of gastrointestinal illness following the challenge (27). We assessed the taxonomic and functional differences between the symptomatic and asymptomatic outcome groups using whole-metagenome shotgun sequencing and bioinformatic analyses. We further used longitudinal data from three infected and symptomatic individuals to determine changes in microbiome structure and function following the challenge and documented the time until recovery to a “baseline” state for the symptomatic individuals. Taken together, the data from this study provide important new insights into the effect of norovirus infection on human gut microbiome structure, function, and response.

## RESULTS

### Human subject responses.

The nine subjects chosen for this study ranged in age from 19 to 27 years and represented both sexes and various ethnicities, factors not linked to symptomatic outcome (see Table S1 in the supplemental material). Of the nine subjects, four developed symptoms of gastrointestinal illness (defined as vomiting and/or diarrhea), while five subjects were asymptomatic. Stool samples were collected from all subjects the day before the norovirus challenge (day 0 [T = 0]) and processed for DNA extraction and norovirus titer measurements. Three of the symptomatic individuals were tracked for up to 33 days following infection, with periodic stool collections (Table S1).

10.1128/mBio.02634-20.6 TABLE S1 Study subject demographics, sample sequencing, alpha diversity, read mapping, assembly metrics, and norovirus titer data (where available) for all samples used in this study. The percentage of trimmed and quality-filtered reads mapping to the corresponding assembly are shown pre- and post-human DNA filtering. “# OTUs” represents the number of taxonomically distinct 16S rRNA sequences extracted from unassembled metagenomes. The outcome for each individual is provided in the “utcome group” column (A, asymptomatic; S, symptomatic). Download Table S1, XLSX file, 0.1 MB. Copyright © 2020 Patin et al. 2020 Patin et al. This content is distributed under the terms of the Creative Commons Attribution 4.0 International license.

### Metagenome sequence coverage.

Metagenome sequencing effort per sample (i.e., number of reads) pre- and post-quality control are provided in Table S1. Nonpareil 3.0 analysis, a tool to assess the fraction of the extracted DNA that was sequenced based on the level of redundancy among sequenced reads (28), showed estimated metagenome coverage values of >80% for all samples (see Fig. S1), indicating that our sequencing effort was large enough to draw robust conclusions from the data sets. Prechallenge metagenome alpha diversities were not significantly different between symptomatic and asymptomatic individuals based on nonpareil sequence diversity index (N_d_) values, a metric based on sequence diversity (*t* test, *P* = 0.23) (Fig. S1A; Table S1), and there were no differences over time in the three symptomatic individuals (Fig. S1B to D). Stool viral titers for the study cohort were previously published (9), and we provide data from the three symptomatic individuals (15, 36, and 37) with longitudinal microbiome data in Table S1. Titers spiked following the challenge on day 1 and gradually decreased over time, falling below detection levels between 20 and 34 days following the infection.

10.1128/mBio.02634-20.1 FIG S1 Nonpareil curves showing estimated metagenome coverage as a function of the sequencing effort applied for three symptomatic individuals for all baseline microbiomes (A) and over the study time course (B to D). (A) All microbiomes at time zero and microbiomes for individual 15 (B), individual 36 (C), and individual 37 (D). In panels B to D, time points are given as recovery 1 to 4, because the sampling days differed for each individual. The arrows indicate the inflection point of the corresponding curve, the natural log of which is the nonpareil sequence diversity index (N_d_) (values for all samples provided in Table S1). Download FIG S1, PDF file, 1.0 MB. Copyright © 2020 Patin et al. 2020 Patin et al. This content is distributed under the terms of the Creative Commons Attribution 4.0 International license.

### Prechallenge microbiomes of symptomatic and asymptomatic individuals.

**FIG 1:**
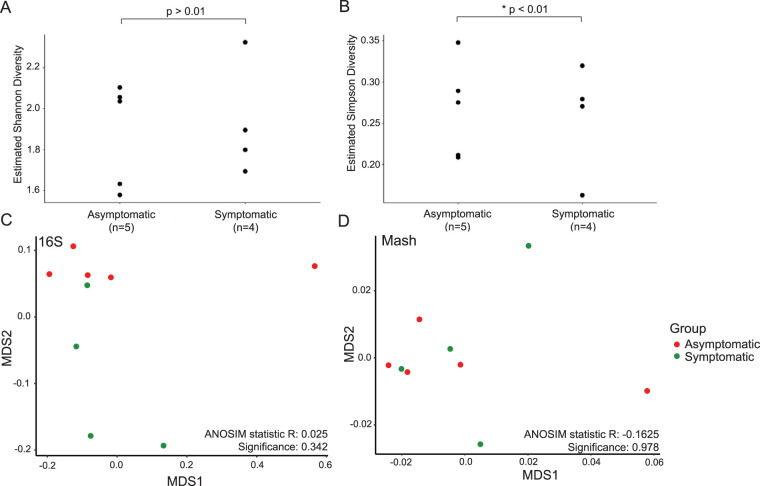
Alpha and beta diversities of prechallenge microbiomes were different between asymptomatic and symptomatic individuals. Alpha diversity of asymptomatic microbiomes showed (A) lower Shannon diversity (not significant) and (B) significantly higher Simpson diversity than for symptomatic microbiomes (*, *P* < 0.01) based on extracted 16S rRNA reads. Microbiomes show some separation according to Bray-Curtis distances of extracted 16S rRNA genes (C) and Mash distances (D) in NMDS plots. ANOSIM test results are provided for each NMDS.

**FIG 2:**
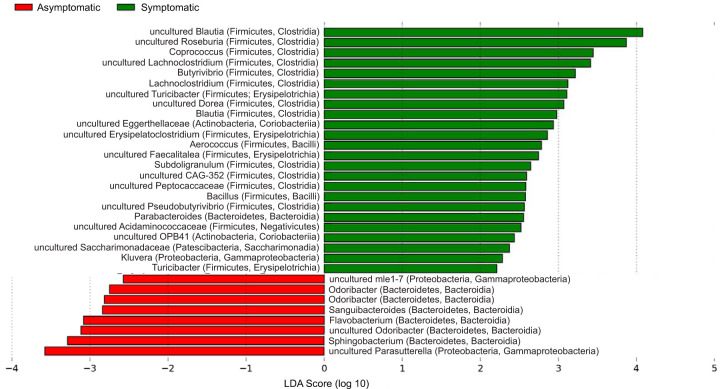
Taxa differentially abundant between symptomatic and asymptomatic individuals at the prechallenge time point (T = 0) using 16S rRNA reads extracted from metagenomes. Taxa enriched in the asymptomatic baseline microbiomes were largely members of the class *Bacteroidia* (phylum *Bacteroidetes*) but also included two gammaproteobacterial taxa. Most of the taxa enriched in the symptomatic microbiomes belonged to the class *Clostridia* in the phylum *Firmicutes*. Each taxon is labeled at the highest resolved taxonomic level, followed by phylum and class in parentheses. The linear discriminant analysis (LDA) score (log 10) of each taxon is represented by the horizontal bars, with red and green bars indicating taxa enriched in microbiomes from asymptomatic and symptomatic study subjects, respectively.

All subjects in this study were positive secretors for the H type 1 HBGA carbohydrate; blood group was therefore not a confounding variable in the comparison of symptomatic and asymptomatic individuals. Prechallenge microbiomes of asymptomatic and symptomatic individuals differed by both alpha and beta diversity metrics calculated using extracted 16S rRNA genes (Fig. 1). Two alpha diversity metrics, the Shannon and Simpson diversity indices, and the variance of each among replicates were calculated (29). While both metrics account for community evenness and richness, they differ mathematically in the calculations. Specifically, the Simpson index places less weight on lower abundance taxa than Shannon entropy. Microbiomes of symptomatic subjects had slightly higher, but not significantly different, Shannon diversity as well as lower Simpson diversity than microbiomes of asymptomatic subjects (Fig. 1A and B). Microbiomes also clustered separately by infection outcome based on beta diversity (see Fig. S2B), although not by k-mer composition (Fig. 1C). Several individual taxa differed significantly in relative abundance between the two outcome groups according to extracted 16S rRNA genes (Fig. 2). Symptomatic individuals featured relatively more species of *Firmicutes*, particularly in the order *Clostridia*, and relatively fewer species of *Bacteroidetes*, particularly in the order *Bacteroidia*, than asymptomatic individuals. Taxa enriched in the asymptomatic baseline microbiomes also included four *Betaproteobacteria*: three members of the genus *Parasutterella* and one in the family *Nitrosomonadaceae*. When all metagenome reads were taxonomically classified, only seven taxa were found be significantly differentially abundant (Fig. S2). One taxon (*Odoribacter* sp.) was enriched in the asymptomatic microbiomes according to both approaches. Overall, both methods identified a rather small number of differentially abundant taxa between symptomatic and asymptomatic samples.

10.1128/mBio.02634-20.2 FIG S2 Taxa differentially abundant between symptomatic and asymptomatic individuals at the prechallenge time point (T = 0) as determined by taxonomic classification of metagenome reads using Kraken 2. The linear discriminant analysis (LDA) score (log 10) of each taxon is represented by the horizontal bars, with red and green bars indicating taxa enriched in microbiomes from asymptomatic and symptomatic study subjects, respectively. Download FIG S2, PDF file, 0.2 MB. Copyright © 2020 Patin et al. 2020 Patin et al. This content is distributed under the terms of the Creative Commons Attribution 4.0 International license.

Genome binning of individually assembled metagenomes produced 665 total metagenome-assembled genomes (MAGs), of which 151 were above the quality threshold (see Materials and Methods). Dereplication yielded 67 MAGs that were used for downstream analyses (see Table S2), including read mapping and calculation of the truncated average sequencing depth (TAD80), a proxy for relative abundance.

10.1128/mBio.02634-20.7 TABLE S2 Taxonomy and per-sample read counts for each taxon with significantly differential abundance between symptomatic and asymptomatic prechallenge (T = 0) microbiomes. These results are based on a LEfSe analysis of the 16S rRNA gene sequences extracted from metagenomes. Download Table S2, XLSX file, 0.1 MB. Copyright © 2020 Patin et al. 2020 Patin et al. This content is distributed under the terms of the Creative Commons Attribution 4.0 International license.

**FIG 3:**
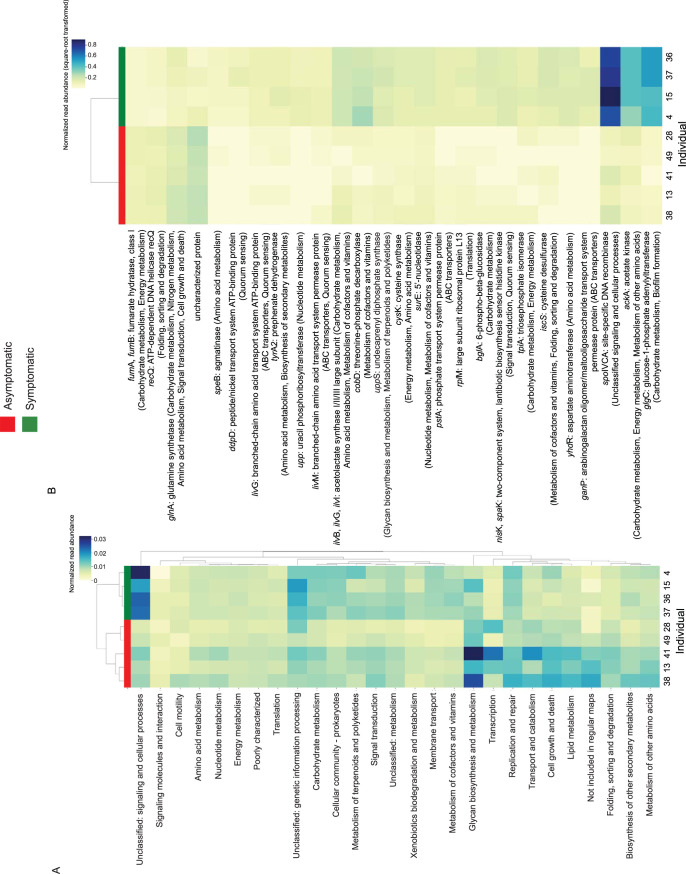
Relative abundances of processes and genes that differentiated the asymptomatic and symptomatic individuals. (A) Representation of each second-order KEGG category. (B) All 26 significantly different individual genes (KOs) between the two outcome groups.

Gene functional annotations of the microbiomes before challenge differentiated asymptomatic from symptomatic individuals (Fig. 3; see also Tables S4 and S5). Of the 290 lowest-level KEGG categorical groupings (subgroup 2), 28 exhibited significantly different relative abundances between the two outcome groups. These included metabolism pathways of compounds found in extracellular polymeric substances (EPS), such as glycans and sphingolipids (Fig. 3), as well broader categories such as RNA degradation (the category of highest significance) (Table S4) and ABC transporters (Fig. 3), which are involved in a variety of cellular functions.

In addition, 26 of 1,936 individual genes were significantly differentially abundant between the microbiomes of symptomatic and asymptomatic study subjects (Fig. 3B; Table S5). Seven of these genes are involved in carbohydrate metabolism biochemical pathways, and an additional four are involved in glycan metabolism and biosynthesis (Table S5). The most differentially abundant genes encoded an acetate kinase (*ackA*), a fumarate hydratase (*fumA* and *fumB*), a peptide/nickel transport system ATP-binding protein, and a DNA recombinase (Table S5).

### Temporal microbiome shifts following viral challenge.

Three symptomatic subjects (individuals 15, 36, and 37) were sampled following the infection challenge, with sampling intervals ranging from 1 to 8 days, for a period of up to 33 days postchallenge (Table S1). These longitudinal sample sets were used to assess microbiome changes over time following a norovirus infection.

Large fractions of contaminant human reads observed in shotgun metagenomes obtained from fecal samples have been a concern in studies addressing the gut microbiome during diarrheal episodes (30–32). In our data sets, human reads comprised a very small proportion of the sequenced metagenome. The fraction they represented increased slightly (by up to 0.06%, in total) during the infection but never exceeded 0.1% of all reads (Table S1).

**FIG 4:**
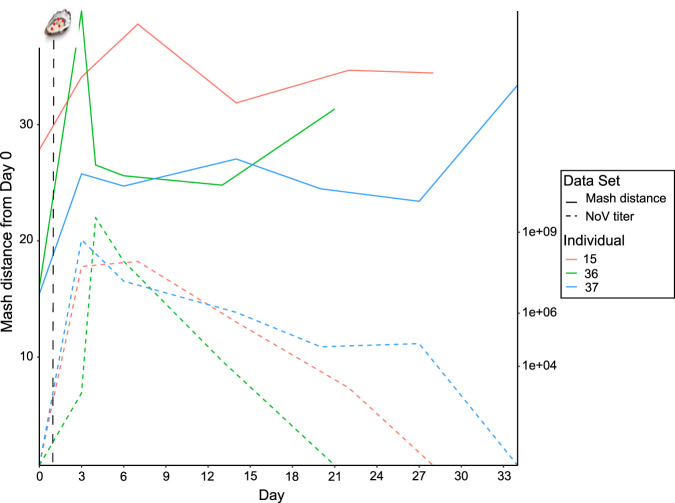
Whole-community similarity over the infection period, with the challenge administered on day 1 (black dashed line). Solid lines represent the Mash distance of whole metagenomes over time and reveal a shift away from time zero (baseline) coinciding with the increase in norovirus titer (dashed lines). As virus titers decrease, Mash distances initially drop, with those for two of three individuals rising again in later time points.

On average, gut microbiome Shannon diversity decreased immediately following the infection challenge and fluctuated throughout the time series (see Fig. S3A). Simpson diversity was more stable, with a slight increase following infection, suggesting community evenness was not substantially altered (Fig. S3B). Metagenome k-mer compositions of three symptomatic individuals were highly distinct, but each microbiome showed shifts away from the prechallenge state between days 2 and 7, with a subsequent return to a similar composition to that in the prechallenge state (Fig. S3C). This analysis should be interpreted with caution, as it did not include a self-versus-self Mash distance calculation to account for inherent stochasticity in sequence data sets (self-versus-self distance must be represented as zero in an ordination plot). When day 0 distance from day 0 was calculated by splitting the data set in half and comparing the two halves, the same shift away from baseline was also observed as a function of overall k-mer distance from day 0 (prechallenge) (Fig. 4), although the temporal patterns were slightly different. Relative to day 0 self-versus-self distance, metagenomes k-mer distances showed an increase immediately following infection with close, but not complete, return to the prechallenge state by the end of the time course. The shifts corresponded to the spike in viral titers (Fig. 4), which began to decrease by days 7 to 10 postchallenge but remained relatively high for several days following the challenge. Asymptomatic individuals also experienced shifts in their microbiome following the challenge, but these were generally of a lower magnitude than those of symptomatic individuals (see Fig. S4).

10.1128/mBio.02634-20.3 FIG S3 Temporal differences in alpha diversity and Mash distance of three symptomatic individuals. Alpha diversities of infected individual microbiomes over time show lower Shannon diversity (A) and similar Simpson diversity (B) at the end of the time course relative to those at the prechallenge state (day 0). Time points were grouped as follows: 0, day 0; 1, day 3; 2, days 4, 6, and 7; 3, days 13 and 14; 4, days 20, 21, and 22; 5, days 27 and 28; 6, day 34. Only one individual was sampled at day 34 (time point 6). (C) Metagenome distances from the metagenome of the same individual at prechallenge (day 0) based on Mash distances (k-mer size = 25). Note that metagenomes of symptomatic individuals shift following the challenge and subsequently returned close to baselines. Download FIG S3, PDF file, 0.9 MB. Copyright © 2020 Patin et al. 2020 Patin et al. This content is distributed under the terms of the Creative Commons Attribution 4.0 International license.

10.1128/mBio.02634-20.4 FIG S4 Slopes of the metagenome Mash distance lines between prechallenge (day 0) and the first postchallenge time point for asymptomatic and symptomatic individuals. Symptomatic individuals showed higher rates of change, but the difference was not statistically significant (*t* test *P* value > 0.05). Download FIG S4, PDF file, 0.8 MB. Copyright © 2020 Patin et al. 2020 Patin et al. This content is distributed under the terms of the Creative Commons Attribution 4.0 International license.

### Functional gene and MAG abundance shifts over time.

MAGs were assigned to two groups based on the change in coverage immediately following the challenge: those that increased in relative abundance and those that decreased or remained the same. Both groups featured a range of temporal responses, with some MAGs returning to prechallenge levels relatively quickly, while others never fully recovered (see Fig. S5). Several MAGs featured different responses depending on the individual and were excluded from the taxonomic and functional gene analyses.

10.1128/mBio.02634-20.5 FIG S5 Relative abundance of recovered MAGs in the three symptomatic individuals over time. Abundance was estimated as normalized TAD80 (coverage) values. MAGs were grouped based on their abundance patterns, and the two panels show MAGs that increased (A) or decreased (B) or were unaltered in coverage following the viral challenge at day 1, represented by the dashed line, for each of the three individuals (in different colors). The solid lines represent the median TAD80 values for all MAGs, and the shaded areas represent the 95% confidence intervals around the median. Download FIG S5, PDF file, 1.0 MB. Copyright © 2020 Patin et al. 2020 Patin et al. This content is distributed under the terms of the Creative Commons Attribution 4.0 International license.

MAGs belonging to the “increase” group were exclusively assigned to the phylum *Firmicutes*, with seven in the order *Clostridiales* and two in the order *Negativicutes*. In contrast, the “decrease” group featured members of the phyla *Bacteroidetes* (12) and *Proteobacteria* (2) (see Table S3). The closest relatives of these MAGs included Eubacterium coprostanoligenes and Gemmiger formicillis, found in both groups, as well as Eubacterium rectale, Blautia wexlerae, Bacillus cereus, and Ruminococcus bicirculans, found only in the “increase” group, and Prevotella stercorea, Faecalibacterium prausnitzii, and Shigella sonnei, found only in the “decrease” group (Table S3).

10.1128/mBio.02634-20.8 TABLE S3 Sixty-seven high-quality dereplicated MAGs generated from all metagenomes and their corresponding response immediately following the infection (increase versus decrease in TAD80). Response group was “N/A” if the MAG showed different behavior in different individuals. Taxonomy provided by MiGA using the NCBI Type Material database, and closest known relative by average amino acid identity (AAI) is shown in columns 3 and 4. All MAGs belonged to the domain *Bacteria*. Download Table S3, XLSX file, 0.1 MB. Copyright © 2020 Patin et al. 2020 Patin et al. This content is distributed under the terms of the Creative Commons Attribution 4.0 International license.

10.1128/mBio.02634-20.9 TABLE S4 All biochemical processes at the “s ubgroup 2” hierarchical category level that were significantly different between asymptomatic and symptomatic prechallenge (T = 0) microbiomes. Download Table S4, XLSX file, 0.1 MB. Copyright © 2020 Patin et al. 2020 Patin et al. This content is distributed under the terms of the Creative Commons Attribution 4.0 International license.

10.1128/mBio.02634-20.10 TABLE S5 Genes (KOs) that were significantly different (corrected *P* < 0.05) in normalized gene abundances between asymptomatic and symptomatic prechallenge (T = 0) microbiomes, and between the “increase” and “decrease” MAGS. KEGG categories and subcategories for each gene are also provided, along with corrected *P* values and fold changes. In cases where genes belong to multiple categories, only one is shown for the sake of clarity. Fold change is provided as the ratio of average normalized reads mapping to each gene in the symptomatic versus asymptomatic metagenomes, and in the increase versus decrease MAGs. Only one gene (K01676, fumarate hydratase) was significantly different in both data sets, and this gene is marked by an asterisk. Download Table S5, XLSX file, 0.1 MB. Copyright © 2020 Patin et al. 2020 Patin et al. This content is distributed under the terms of the Creative Commons Attribution 4.0 International license.

**FIG 5:**
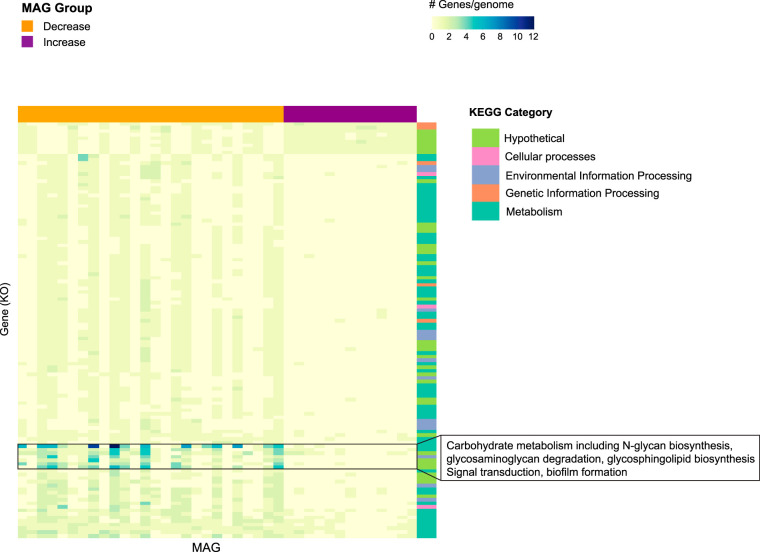
Genes that differed significantly (corrected *P* < 0.05) in copy number/genome between MAGs that increased (purple) or decreased (orange) following the viral challenge. Functions enriched in “decrease” MAGs included proteins involved in carbohydrate metabolism and signal transduction, while functions enriched in “increase” MAGs were largely hypothetical. The highest-level functional category is provided by a color bar on the *y* axis (“hypothetical” refers to uncategorized KEGG annotations, i.e., “not included in pathway or BRITE”).

Of 3,645 genes tested, 116 differed significantly (corrected *P* < 0.05) in abundance between the “increase” and “decrease” MAGs (Table S5; Fig. 5). These genes were part of 17 KEGG subcategories (subgroup 1), including carbohydrate metabolism (33 genes) and amino acid metabolism (15 genes) (Fig. 5). They also include genes encoding proteins involved in transmembrane sensing and export, metabolism of cofactors and vitamins, and a fumarate hydratase that was also significantly different in abundance between the metagenomes of asymptomatic and symptomatic subjects.

## DISCUSSION

Despite its prevalence and widespread public health impacts, the effects of norovirus infection on the human gut microbiome remain poorly understood. As a primarily enteric pathogen, the virus enters epithelial cells in the digestive tract and initiates a course of infection that is generally acute and self-limiting but, in rare cases, can lead to debilitating symptoms. However, a proportion of infected individuals exhibit no symptoms of gastrointestinal infection, an outcome distinct from resistance to infection (33, 34). Several lines of evidence suggest that viral pathogenicity is affected by the number and types of microbes encountered by the virus in the intestine (14, 15, 20, 35, 36), as is the case with several other enteric pathogens (37, 38). Our study provides some of the first data to support this hypothesis using a human challenge-response study design and deep metagenomic sequencing to assess gut microbiome correlations in symptomatic versus asymptomatic outcomes and response to infection.

Human gut microbiomes are extremely diverse and complex, with high levels of interindividual variability (39). We used several different metrics to assess broad-scale differences in microbiome composition between asymptomatic and symptomatic individuals and found small, but significant, differences between the two outcome groups. Mash distance, a measure of overall metagenome nucleotide composition similarity, was not an effective predictor of infection outcome (Fig. 1D), and alpha and beta diversity were not significantly different between outcome groups (Fig. 1A to C). This is likely due to the high level of interindividual variability among human microbiomes, which are influenced by multiple factors, including diet, environmental exposure, and physiological conditions (39). Nevertheless, several taxa differed significantly between the two outcome groups (Fig. 2; see Fig. S2 in the supplemental material). Different taxa were identified using two different methods of estimating community composition (extracted 16S rRNA genes versus k-mer-based taxonomic classification of all reads); this discrepancy is likely due to inherent limitations and biases in both methods, e.g., the use of different reference databases. Assessing the reliability of each method is beyond the scope of this study; we therefore focus here on the 16S-based analysis. The higher levels of *Bacteroidetes* in the asymptomatic individuals, particularly members of the class *Bacteroidia*, suggested that these taxa confer some advantage in resisting enteric viral infection or neutralizing its pathogenicity and symptoms. Consistent with these findings, higher levels of *Bacteroidetes* have been linked to gut microbiomes from individuals who are healthy and have recovered from a range of acute enteric infections (40, 41), although there are different outcomes depending on the exact genera and species present. Interestingly, members of the family *Enterobacteriaceae*, which have been shown to express HBGA-like molecules on their cell membranes and thus facilitate viral attachment to human B cells (14, 19), were not among the differentially abundant taxa. Their absence indicates that the role of this family in norovirus infection may not be essential. Noroviruses can effectively bind to a range of microbial taxa (21), but in some cases, the efficiency is dependent upon the growth medium (35), suggesting nutrient availability may play an additional role in binding.

The separation of symptomatic and asymptomatic individuals was also evident in their different functional gene potentials (Fig. 2; Fig. S3C). Because some biochemical processes encompass genes with potentially wide diversity among individual human microbiomes, we assessed differences in relative abundances at both the functional category and individual gene level. Numerous functional categories, pathways, and genes were differentially enriched between the two groups. Many of these are likely not directly involved in immune function or response to viral pathogenicity; rather, they are different because they are associated with taxa that function in defense or resistance to HuNoV (a so-called “hitchhiker effect”). Moreover, some of these genes may reflect the gene content of taxa that are differentially abundant between the two subject groups by chance or another reason not related to the biochemical function in question. To validate the genes identified here as part of the protective mechanism(s) versus hitchhiker effects would require experimental testing. Nonetheless, it is possible to propose reasonable hypotheses based on the current understanding of norovirus tropism and the (predicted) functions found to differentiate the microbiomes of symptomatic study subjects versus those of asymptomatic subjects. More specifically, processes, including glycan biosynthesis and metabolism, lipid metabolism, and signaling and cellular processes, were differentially enriched between the two groups (Fig. 3A). Of the 26 significantly differently abundant genes, several are involved in multiple biochemical pathways, while others are specific to one process (Fig. 3B). Moreover, both genes and categories include uncharacterized processes. Nevertheless, certain patterns may point to underlying biological mechanisms of an asymptomatic infectious state. For example, two of the most differentially enriched genes in symptomatic microbiomes are involved in carbohydrate metabolism, including the glycogen biosynthesis gene *glgC*. Glycogen is generally considered an intracellular storage polysaccharide (42), and the enrichment of *glgC* may represent more taxa diverting polysaccharides to energy reserves rather than to the biosynthesis of extracellular polymeric substances (EPS), which facilitate viral attachment and subsequent infection of human B cells (14, 19). Noroviruses interact in various ways with outer membrane molecules; for example, a human milk oligosaccharide was found to inhibit the binding of one norovirus genotype to HBGAs (43). It is therefore likely that there exist as-yet-unknown extracellular molecules in the gut microbiome that influence infection outcome, and these patterns are reflected in the microbial functional potential to synthesize and transport glycans, sphingolipids, and lipoproteins.

Norovirus infections can have long-lasting effects on host health and physiology (24), well past the typical 12- to 48-h duration of symptoms. Viral shedding can persist for several weeks postchallenge (8, 24, 34, 44), suggesting the gut microbiome is impacted by viral replication and possibly delaying full recovery. We examined longitudinal stool samples from three infected symptomatic individuals and found that alpha diversity (Fig. S3) and microbiome composition (Fig. 4; Fig. S3C) were altered immediately following the infection. The magnitude of change and the time until a partial return to the prechallenge state varied among the three individuals, but the long-lasting effects were coincident with the persistence of HuNoV in the gut (Fig. 4). Consistent with the duration of the altered microbiome composition, we detected HuNoV up to 26 days following the challenge, with an average 20.7-day duration for three symptomatic individuals (Table S1), and shedding magnitudes were similar to those of asymptomatic individuals, as reported previously (9). Detection of viral RNA in stool has been reported for up to 60 days postchallenge (8, 24), though symptoms generally resolve much sooner. There are conflicting reports in the literature regarding viral shedding duration; some studies showed similar values in asymptomatic and symptomatic individuals (24, 25), while others found significant differences (8, 34). Our results offer further support for the latter pattern. Asymptomatic individuals also experienced microbiome shifts following the challenge, but the rate and magnitude of change was not as dramatic as in symptomatic individuals (Fig. S4), suggesting asymptomatic outcome is accompanied by greater overall microbiome stability.

We evaluated the changes in 67 microbial populations, represented by metagenome-assembled genomes (MAGs), over the course of infection and found a wide variety in response patterns (Fig. S5). MAGs were divided into two groups, those that increased immediately during infection and those that decreased, presumably reflecting taxa that are differentially adapted to changing gut conditions resulting from HuNoV infection. Within each group, there were various patterns of recovery and stability in coverage over time (Fig. S5). These data support the idea of a dynamic nonlinear response to viral perturbation that is dependent on a variety of both host biological and microbiome factors. The populations that decreased following infection were enriched in members of the class *Bacteroidia* (phylum *Bacteroidetes*) relative to those that increased (Table S3), suggesting these taxa are more susceptible to the HuNoV-induced perturbation. The differences in taxonomy were accompanied by differences in biochemical functions, including outer membrane proteins, folate biosynthesis proteins, and polysaccharide biosynthesis/export proteins, all of which were enriched in MAGs that decreased during infection (Fig. 5; Table S5). These functions may not be involved in HuNoV infection of human cells but rather may be part of the biochemical repertoire of taxa that provide infection resistance. *Bacteroidia* were also enriched in the prechallenge microbiomes of asymptomatic individuals (Fig. 2), suggesting a link between taxa unlikely to facilitate viral infection and those that respond negatively once infection is initiated. The underlying assumption is that if *Bacteroidia* are less likely to express the specific extracellular compounds that mimic HBGAs, and thus confer resistance, they may also be disproportionately affected when the virus successfully initiates infection. In effect, bacteria that have evolved to facilitate viral infection (e.g., *Enterobacteriaceae* or the *Clostridiales* enriched in symptomatic individuals) may also be better adapted to an environment characterized by HuNoV proliferation and the host immune response.

### Conclusions.

In this study, we provide some of the first data on human gut microbiome composition and function in the context of norovirus infection. These results, while based on a small number of subjects and only one strain of human norovirus (genogroup I.1), are nonetheless valuable because the exposure and course of infection are well characterized and because human experiments are challenging to conduct and limited in number. Importantly, we generated a list of high-quality genomes that can be further characterized or used as a reference in future studies to understand resistance to norovirus infection. Characterization of the overall microbiome taxonomic compositions showed important differences in the taxa enriched in the asymptomatic individuals versus those in symptomatic individuals. Furthermore, we generated hypotheses about specific genes and pathways that can be tested in future experiments. These data bring us toward a better understanding of norovirus pathogenicity and how future outbreaks may be controlled, treated, or prevented.

## MATERIALS AND METHODS

### Study design and sample preparation.

The Norwalk virus human challenge study was designed and conducted as described in reference 27, a study whose primary goal was to determine whether heat and pressure treatment of contaminated seafood could inactivate norovirus. The authors concluded this treatment was ineffective; we therefore leveraged samples from subjects administered infected oysters to conduct our own study addressing the effects of norovirus on the human gut microbiome. In the original study, 44 individuals were selected for participation based on specific inclusion and exclusion criteria, including overall good health and low potential to transmit the virus to other susceptible individuals after challenge. Demographic characteristics of all subjects are provided in reference 27. Samples from 9 of these 44 individuals were sequenced as part of the present study based on the patients’ treatment conditions (ingestion of infected but otherwise unmodified oysters). All participants were positive secretors of the H type 1 HBGA carbohydrate. Norwalk virus (genogroup I.1 HuNoV inoculum 8FIIb) RNA was extracted from stool filtrates and quantified by real-time reverse transcription-quantitative PCR (RT-qPCR). Commercial oysters (Crassostrea virginica) were prepared by high hydrostatic pressure processing (HPP) of 400 MPa for 5 min to inactivate any potential pathogens acquired from the harvest area. The HuNoV inoculum was injected into the tissue of multiple batches of three oysters (*Crassostrea virginica*) 3 days prior to the challenge, such that each batch of three oysters contained 1.0 × 10^4^ genomic equivalent copies (GEC) of HuNoV in total. Each study subject ingested one batch of three inoculated oysters, including oyster juice, and also ingested approximately 2.4 g sodium bicarbonate dissolved in water 2 min prior to, and 5 min after, oyster consumption to reduce stomach acidity. Subjects were classified as either symptomatic (*n* = 4) or asymptomatic (*n* = 5) based on the occurrence of gastrointestinal responses, including diarrhea and vomiting. Gut microbiomes (stool samples) of all nine individuals were sampled 1 day prior to the challenge (day 0). Microbiomes of three symptomatic individuals were sampled for up to 33 days following the challenge. An additional symptomatic individual was sampled only once postinfection, 11 days following the challenge. Five asymptomatic individuals were sampled at various time points following the challenge (Table 1).

**TABLE 1:** Longitudinal sampling scheme for nine study subjects infected with HuNoV GI.1, including four symptomatic and five asymptomatic individuals

Subject ID^*a*^	Outcome	Sampling time point (day postchallenge)^*b*^															
		0	2	3	4	5	6	7	12	13	14	20	21	22	27	28	34
4	Symptomatic	X							X								
15	Symptomatic	X		X				X			X			X		X	
36	Symptomatic	X		X	X		X			X			X				
37	Symptomatic	X		X			X				X	X			X		X
13	Asymptomatic	X	X		X												
28	Asymptomatic	X		X													
38	Asymptomatic	X												X			
41	Asymptomatic	X						X									
49	Asymptomatic	X	X			X											

a ID, identifier.

b Time points when stool samples were collected are marked by an X. All individuals provided stool samples at day 0, 1 day prior to the virus challenge.

Study protocols and sample collections for the original Norwalk virus challenge study (27) (ClinicalTrials.gov identifier NCT00674336) were approved by an independent DSMB and the Emory University Institutional Review Board.

### Sample collection, processing, and sequencing.

DNA from stool samples was extracted from a homogenized stool mix using the MO BIO PowerSoil DNA isolation kit and following the standard manual of procedures (MoP) suggested by the Human Microbiome Project (http://hmpdacc.org/resources/tools_protocols.php). The purity and concentration of the DNA were measured using a NanoDrop spectrophotometer (Thermo Fisher Scientific) and the Qubit double stranded DNA (dsDNA) high-sensitivity assay (Invitrogen). Metagenomic libraries were prepared using the Nextera XT DNA library preparation kit (Illumina) according to manufacturer’s instructions, except that the protocol was terminated after isolation of cleaned double-stranded libraries. Library concentrations were determined using a Qubit HS DNA assay and Qubit 2.0 fluorometer (Thermo Fisher Scientific), and samples were run on a high-sensitivity DNA chip using a Bioanalyzer 2100 instrument (Agilent) to determine average library insert sizes. An equimolar mixture of the libraries was sequenced as recommended by the manufacturer on an Illumina HiSeq 2500 instrument (Georgia Institute of Technology Molecular Evolution Core Facility) for 300 cycles (2 × 150-bp paired-end run). Library demultiplexing and adapter trimming were carried out on the instrument.

### Sequencing read quality control.

Paired-end reads were processed and quality filtered with SolexaQA++ (45) with a minimum Phred score of ≥20 for each base and a minimum read length of 50 bp. Filtered reads were run against the latest human genome sequence using BMTagger (46) to identify host DNA contamination. Reads identified as human were quantified and removed from the data for downstream analyses. Abundance-weighted average coverage of the data sets was estimated using Nonpareil (28) with the alignment algorithm and an iterative subsample factor of 0.7.

### 16S rRNA gene extraction and diversity metrics.

Microbiomes were analyzed for differences in alpha and beta diversities using quality-filtered unassembled reads. Nonpareil 3.0 (28) was used to generate curves of estimated average coverage as a function of sequencing depth. The N_d_ value, a metric based on sequence diversity, was calculated for each metagenome. Differences in alpha diversities between asymptomatic and symptomatic baseline microbiomes were tested using a student’s two-tailed *t* test of the sample N_d_ metrics. 16S rRNA gene-carrying reads were extracted from the metagenomes using Parallel-META (47). The resulting matching reads were run against the SILVA 132 SSU Ref NR 99 database (48) using vsearch (49). Tables with assigned taxonomy and corresponding counts were used to compare the diversities of baseline microbiomes for asymptomatic and symptomatic study subjects as well as assess diversity over time in the symptomatic individuals. Alpha and beta diversity metrics were calculated at the family and sequence variant levels, respectively, using DivNet (29) and plotted using ggplot2 (50). Beta diversity was assessed using the resulting Bray-Curtis distance matrix in nonmetric multidimensional scaling (NMDS) plots with the metaMDS and anosim functions of the vegan package (51) in R.

### Biomarker analysis.

Prechallenge microbiomes of asymptomatic and symptomatic subjects were compared to identify differentially abundant microbial taxa (“biomarkers”) using linear discriminant analysis effect size (LEfSe) (52). This analysis was performed in the following two ways: (i) number of reads mapped to 16S rRNA reads were extracted from metagenomes as described above, and (ii) taxonomic compositions were based on classification of all metagenomic short reads. The latter was performed using Kraken 2 (53), which applies a k-mer-based approach to taxonomically classify short metagenomic reads using the RefSeq database (“standard” prebuilt database includes archaea, bacteria, viral, plasmid, and human reads; https://genome-idx.s3.amazonaws.com/kraken/k2_standard_20200919.tar.gz), followed by Bracken (54), which generates abundance estimations using the taxonomic classifications from Kraken 2. In both cases, the LEfSe analysis was run using raw numbers of reads, not relative abundances, with a per-sample normalization and all-versus-all parameters.

### Metagenome assembly and binning.

Quality-filtered reads were *de novo* assembled using IDBA-UD (55). Genome equivalent values for each metagenome were calculated using MicrobeCensus (56). MaxBin 2.0 (57) was used to bin assembled contigs into metagenome-assembled genomes (MAGs) with a minimum contig length of 2,000 bp. MAGs were generated from each individual metagenome (not a coassembly). Resulting bins were run through CheckM v1.0.3 (58) and the Microbial Genomes Atlas (MiGA) (59) for quality assessment and taxonomic assignment, respectively. Each MAG was assigned a quality score defined as completion minus five times the estimated contamination, and only MAGs with a quality score of >50 were retained for further analysis. To remove redundant bins (i.e., genomes from different samples representing the same microbial taxon), MAGs were dereplicated in a two-step clustering process using dRep (60) using a 95% average nucleotide identity (ANI) threshold for clustering. When multiple MAGs were present in a secondary cluster, the highest-quality MAG was chosen as a representative to be used for subsequent analyses. The final list of dereplicated high-quality MAGs and their taxonomy and closest relatives by amino acid identity (AAI) are shown in Table S3 in the supplemental material, and sequences are available on NCBI under BioProject PRJNA645402.

### Mash distances.

To complement the taxonomy-based community composition comparisons among microbiomes, we also performed k-mer-based assessments of unassembled microbiomes. Overall similarities of metagenomic nucleotide compositions were calculated using Mash (61). To compare baseline microbiomes of asymptomatic (5) and symptomatic (4) individuals, reference sketches were generated for all quality-controlled and filtered reads from the prechallenge (time zero) time point using a k-mer size of 25. Sketches were combined within each outcome group and used to generate an all-versus-all distance matrix.

To compare metagenomes from the three symptomatic individuals over time, reads of a metagenomic data set were first randomly divided into two equal-size files so that prechallenge samples (T_0_) could be self-compared by running one half of the reads against the other half. The resulting files (half-read files) were used to generate reference sketches and an all-versus-all distance matrix, with the T_0_ half-read files run against each other for a starting distance value. Mash distances from the symptomatic individuals were also used to generate a line plot in R showing change in distance over time.

Full read files were used to generate a Mash distance matrix, which was used for an NMDS analysis and an analysis of similarity (ANOSIM) using the metaMDS and anosim functions, respectively, of the vegan library in R (51). Self-versus-self distances were assumed to be zero in this analysis.

### MAG coverage and temporal analyses.

Coverage of each MAG in each sample was calculated by estimating sequencing depth per position using Bowtie 2 (62) with default settings for read mapping, bedtools (63) for coverage estimation, and averaging the central 80% of the distribution, which removes the highest 10% and lowest 10% of outlier positions in terms of coverage (here referred to as truncated average depth [TAD80]). TAD80 values were normalized by the genome equivalent of the corresponding metagenome. Relative abundance of each MAG in each metagenome was calculated as the raw TAD (not the TAD80) multiplied by the MAG size (in base pairs), all divided by the total number of base pairs in the metagenome.

MAGs with an average TAD80 value greater than 0.01 (approximate relative abundance of 1% of the total community) from three symptomatic individuals were analyzed for temporal changes. MAGs were grouped by the change in TAD80 (increase versus decrease) from prechallenge to the first postchallenge time point. MAGs grouped by individual were plotted in Seaborn, with lines representing the median TAD80 values and shaded areas representing the 95% confidence intervals.

MAGs were grouped based on their change in TAD80 from the baseline to the first time point following the challenge as either “increase” or “decrease” depending on the response. MAGs that increased in one individual and decreased in a different individual were excluded from the groups because their response was not consistent. The taxonomy and closest relative (based on average amino acid identity [AAI]) of the MAGs belonging to each group are shown in Table S3.

### Functional annotation.

KEGG gene functional annotations were assigned and quantified for each prechallenge metagenome. Open reading frames were generated using Prodigal (64) and clustered with MeShClust (65) at 90% nucleotide identity. The longest sequence from each cluster was extracted using a custom Python script, and these representative sequences were run against the KEGG ortholog profile hidden Markov models (HMMs) (KOfams) using KofamScan with the “prokaryote” database (66). The parameter “-f mapper” was applied to provide only the most confident annotations (those assigned an individual KO). Orthologues were matched to their corresponding functions using a parsed version of the ko00001.keg database text file (https://github.com/edgraham/GhostKoalaParser), which provides a three-tiered hierarchical categorization of each gene, here referred to as “group,” “subgroup 1,” and “subgroup 2.” Sequence coverage of each gene was generated by mapping metagenomic short reads against each one using Magic-BLAST (67). The Magic-BLAST output was filtered to include only the best match for each read with a ratio of alignment length to read length of 0.7 and a minimum read length of 70 bp. Read counts were normalized by genome equivalent value of the corresponding metagenome in order to provide the relative abundance of the gene to which the reads were mapped (i.e., what fraction of total cells/genomes encode the gene of interest).

The normalized gene counts were used to test for significantly different functions and run hierarchical clustering analyses. KofamScan outputs were grouped by the three hierarchical categories as well as by individual KO number (gene). At the highest categorical level (group), the categories human diseases, organismal systems, cellular community–eukaryotes, and BRITE hierarchies were removed before performing the statistical analyses. The first three groups are not relevant to microbial gene functions, and the fourth provides a different hierarchical categorization scheme for the same annotations that was redundant. Each gene and highest-resolution category (subgroup 2) were tested for significantly different abundance between symptomatic and asymptomatic individuals by running a two-tailed Student’s *t* test on the normalized read counts. A Benjamini-Hochberg multiple-test correction for false discovery rate was run on the individual KO *P* values due to the large number of tests performed (1,936). Genes were considered to be significantly differentially abundant with a corrected *P* value of less than 0.05. Fold change was calculated as the ratio of mean values from symptomatic to asymptomatic subjects. All significantly different categories (subgroup 2 level) and genes (KO level) are shown in Tables S4 and S5, respectively.

Cluster maps were generated at the subgroup 1 level for all categories, and individual cluster maps were generated for each subgroup 2 group determined to be significantly different in relative abundance (28 total). All cluster analyses were run with the “clustermap” function in the Python library Seaborn (68) using the Ward linkage and Euclidean distance methods.

Comparison of genes between the MAGs that increased in relative abundance following the challenge and those that decreased or remained the same was performed using similar methods as for the asymptomatic/symptomatic comparison mentioned above, with the following differences: each MAG was annotated independently with KofamScan such that each MAG was a sample in the statistical comparison rather than an assembly. The *t* test was run on each KO using the number of hits per gene per MAG as sample values, and a Benjamini-Hochberg multiple-test correction for false discovery rate was run on all KOs due to the large number of tests performed (3,645). Fold change was calculated as the ratio of “increase” group to “decrease” group mean values. A cluster map was generated as described above without clustering the MAGs so that differences between the two groups could be easily visualized but with clustering of genes to show groups of genes with similar patterns of relative abundance (Fig. 5). The complete list of significantly different genes (corrected *P* values < 0.05; 116 total) and their corrected *P* values and fold changes are provided in Table S5.

### Data availability.

Raw reads for all metagenomes are available in NCBI under BioProject PRJNA645402. Custom scripts for bioinformatic and statistical analyses can be found at https://github.com/nvpatin/Norovirus_manuscript. The analyses include formatting vsearch taxonomic assignment outputs for downstream analyses, alpha and beta diversity analyses of the extracted 16S rRNA gene sequences (both prechallenge samples and time series of infected individuals), and comparison and visualization of differentially abundant gene content from KofamScan outputs.
