# Erratum for Patin et al., “The Role of the Gut Microbiome in Resisting Norovirus Infection as Revealed by a Human Challenge Study”

**DOI:** 10.1128/mBio.00285-21

**Published:** 2021-03-16

**Authors:** N. V. Patin, A. Peña-Gonzalez, J. K. Hatt, C. Moe, A. Kirby, K. T. Konstantinidis

**Affiliations:** a School of Biological Sciences, Georgia Institute of Technology, Atlanta, Georgia, USA; b Max Planck Tandem Group in Computational Biology, Department of Biological Sciences, Universidad de los Andes, Bogotá, Colombia; c School of Civil and Environmental Engineering, Georgia Institute of Technology, Atlanta, Georgia, USA; d Rollins School of Public Health, Emory University, Atlanta, Georgia, USA; e Waterborne Disease Prevention Branch, Centers for Disease Control and Prevention, Atlanta, Georgia, USA

## ERRATUM

Volume 11, issue 6, e02634-20, 2020, https://doi.org/10.1128/mBio.02634-20. The third paragraph of the Acknowledgments section should read as follows: “Funding for this study was provided by National Institutes of Allergy and Infectious Diseases grant R01AI137679 and by Colciencias through a doctoral fellowship to A.P.-G. We also acknowledge funding from the USDA NIFA (grant 2005-5110-03271) for the original Norwalk virus human challenge study.” That is, a wrong NIH project number was previously provided (1K01AI103544) for the funding that partially supported this study. The correct project number is R01AI137679.

