# Supplementary figures and images for: The Role of the Gut Microbiome in Resisting Norovirus Infection as Revealed by a Human Challenge Study
Source: mBio. 2020 Nov 17;11(6):e02634-20. doi: 10.1128/mBio.02634-20 (PMC7683401; doi:10.1128/mBio.02634-20)

Figure S1

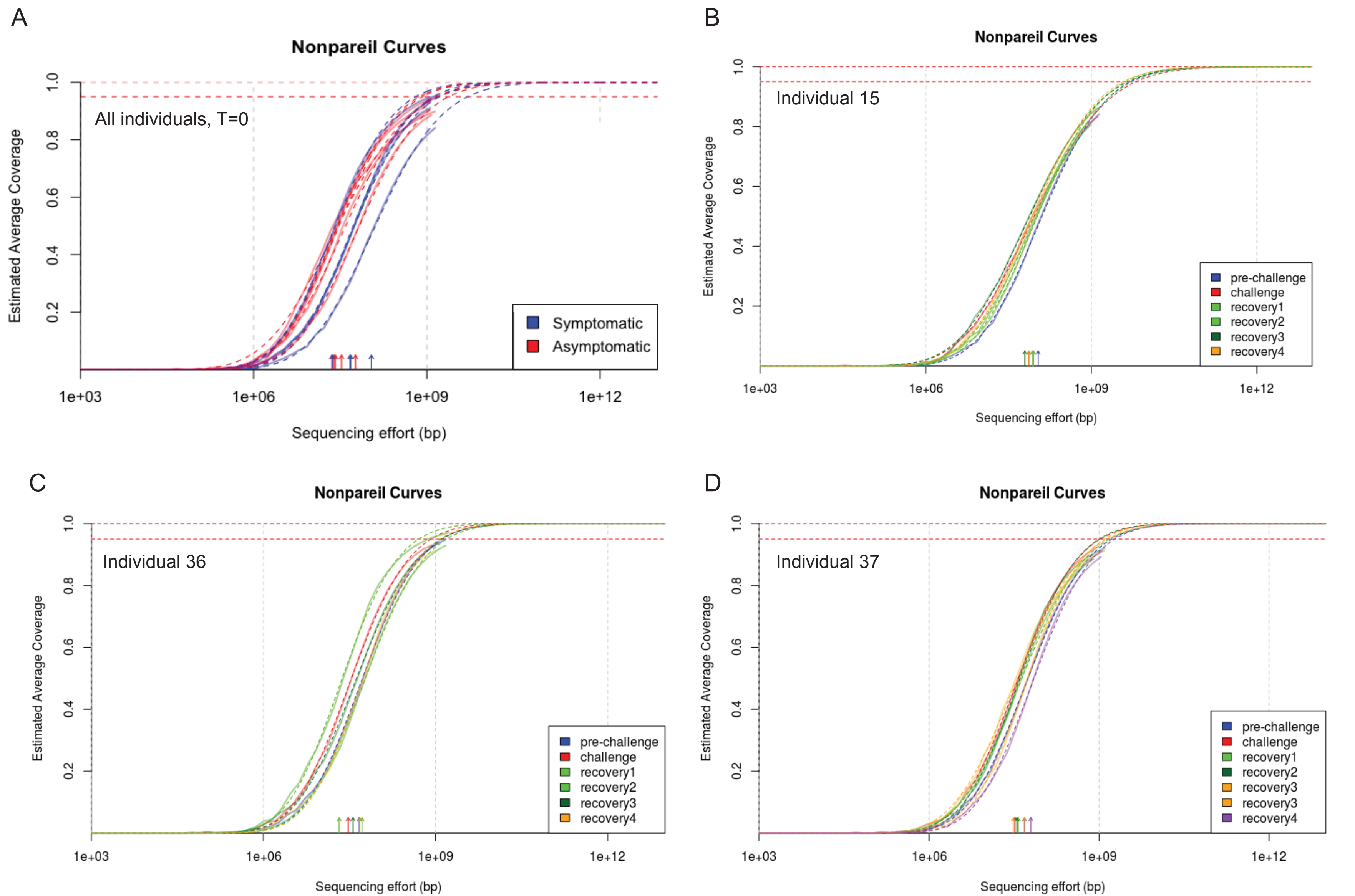

Supplement: FIG S1 [file mBio.02634-20-sf001.pdf]

Figure S2

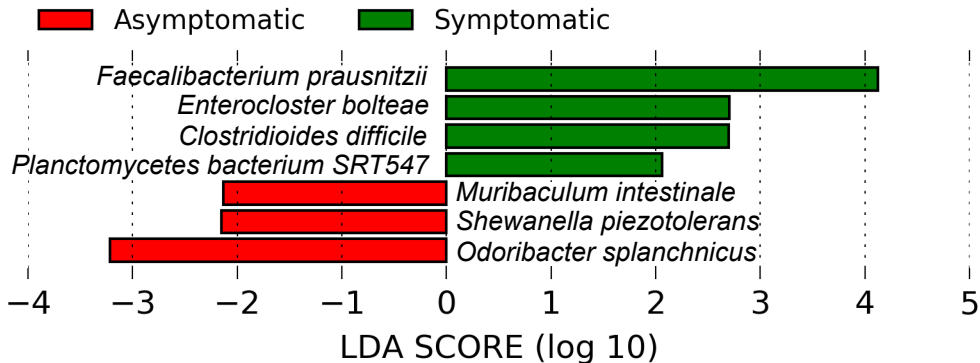

Supplement: FIG S2 [file mBio.02634-20-sf002.pdf]

Figure S3

A

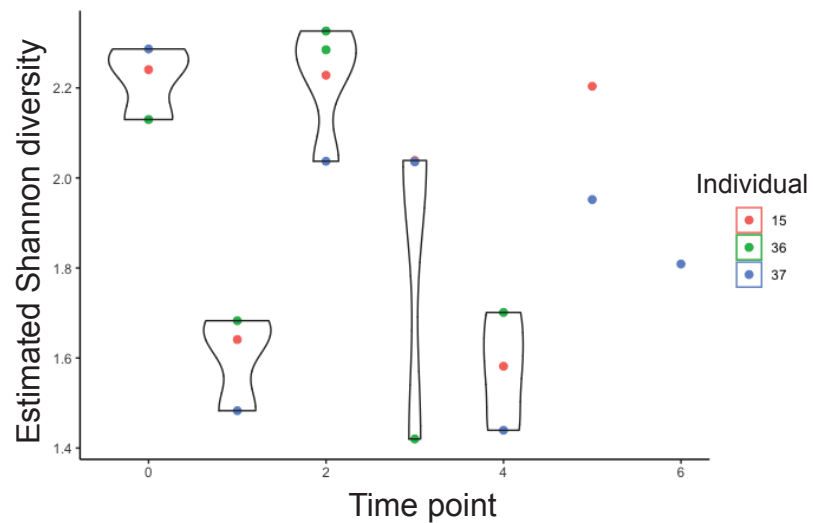

B

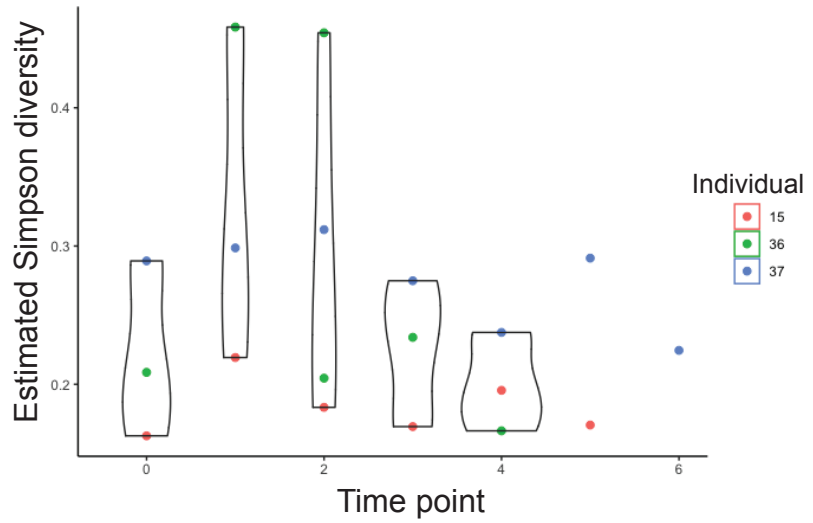

C

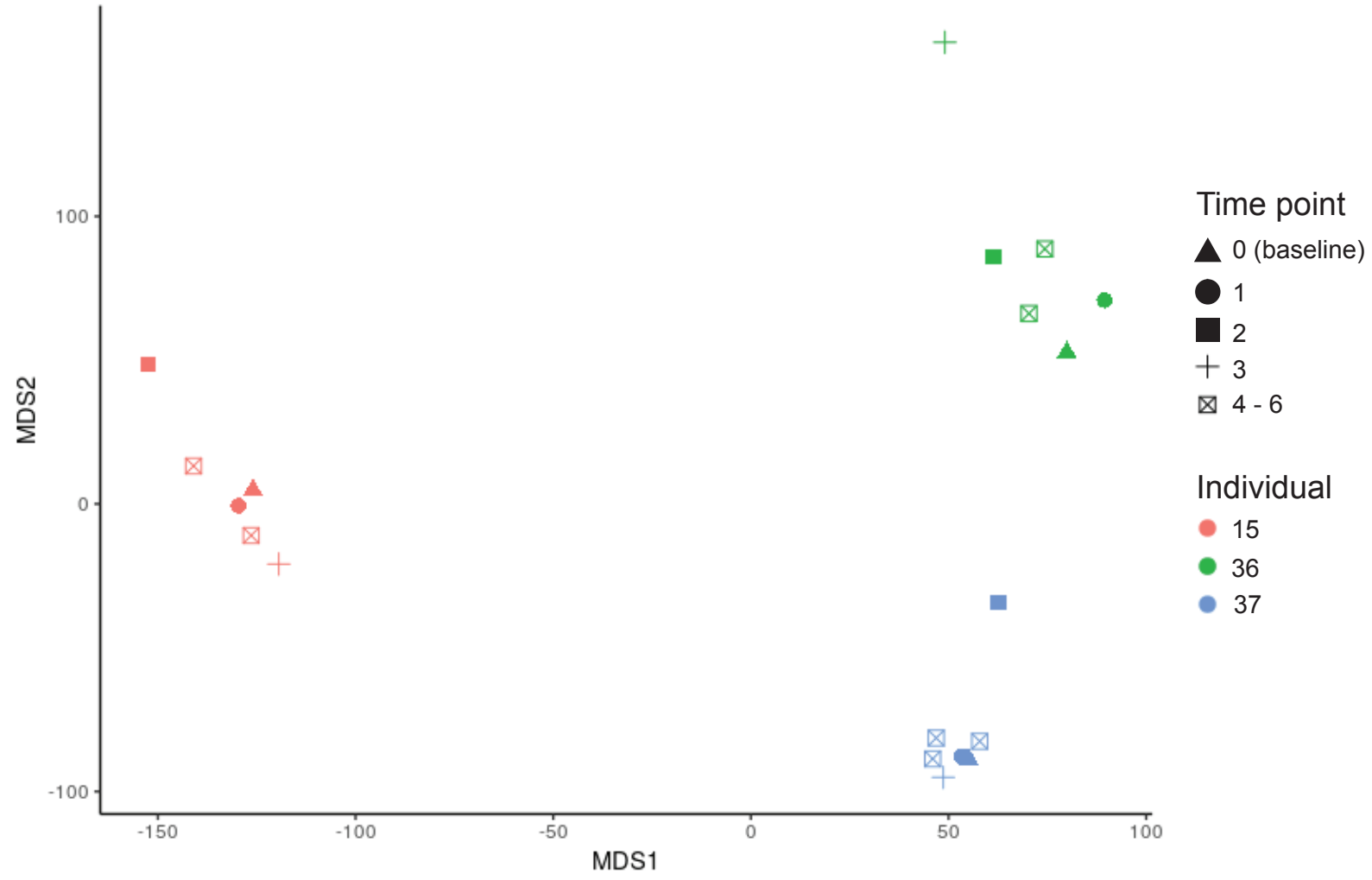

Supplement: FIG S3 [file mBio.02634-20-sf003.pdf]

Figure S4

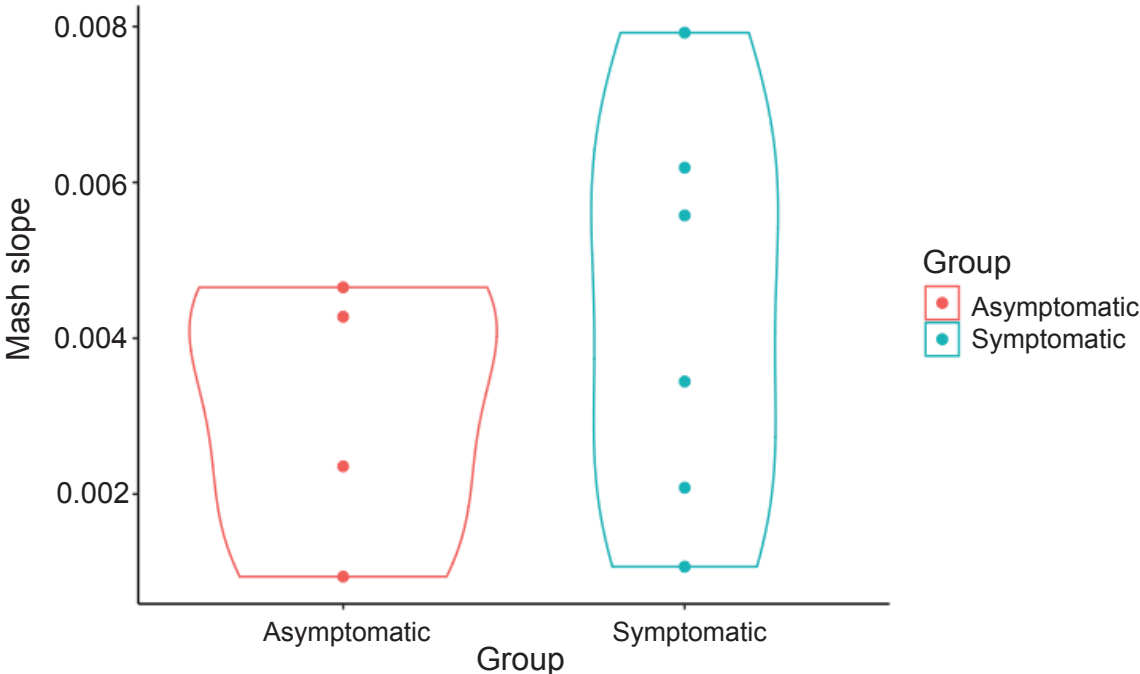

Supplement: FIG S4 [file mBio.02634-20-sf004.pdf]

Figure S5

A

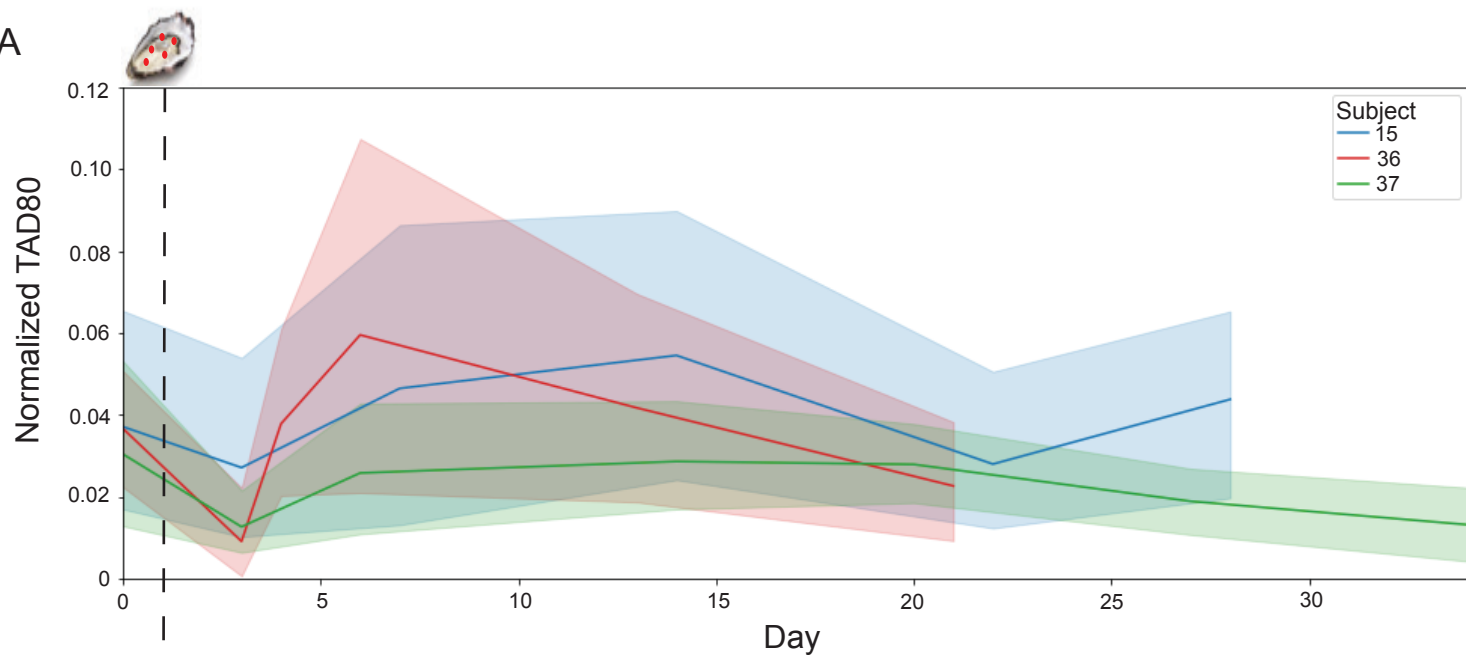

B

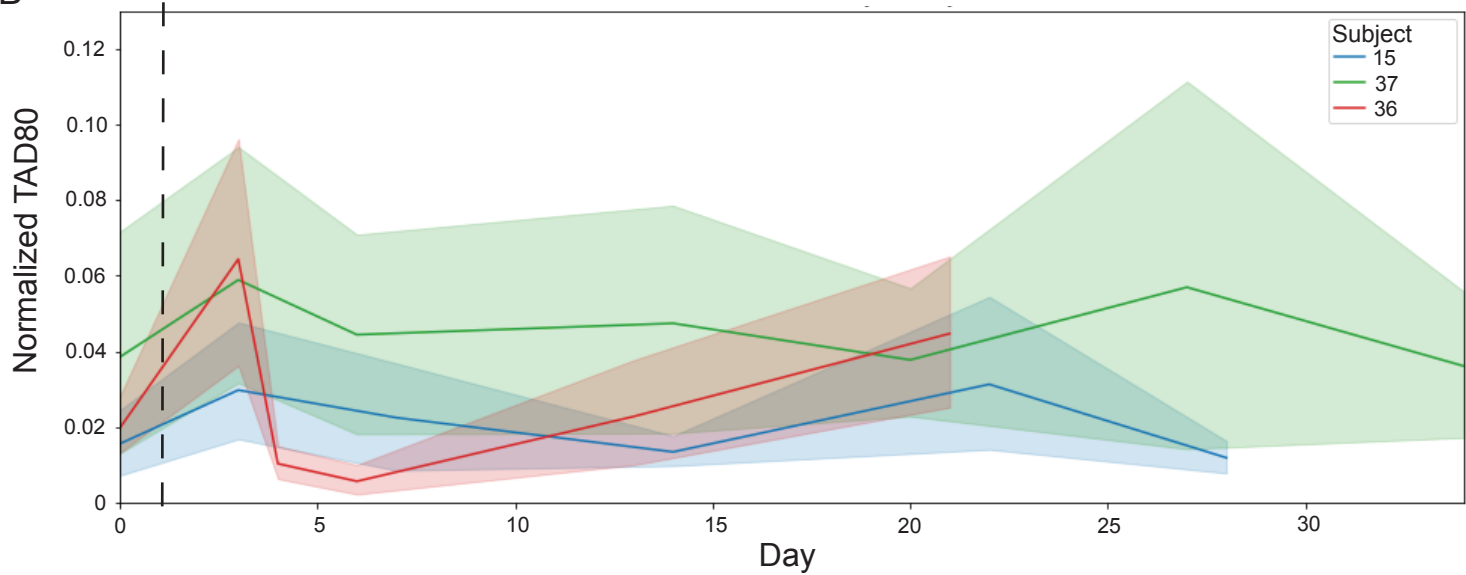

Supplement: FIG S5 [file mBio.02634-20-sf005.pdf]
